# Successful Aging and Subjective Aging: Toward a Framework to Research a Neglected Connection

**DOI:** 10.1093/geront/gnae051

**Published:** 2024-05-20

**Authors:** Serena Sabatini, Fiona Rupprecht, Roman Kaspar, Verena Klusmann, Anna Kornadt, Jana Nikitin, Anton Schönstein, Yannick Stephan, Markus Wettstein, Susanne Wurm, Manfred Diehl, Hans-Werner Wahl

**Affiliations:** Department of Psychological Sciences, Faculty of Health and Medical Sciences, School of Psychology, University of Surrey, Guildford, UK; Department of Developmental and Educational Psychology, Faculty of Psychology, University of Vienna, Vienna, Austria; Charlotte Fresenius University of Psychology, Hamburg, Germany; Department of Health, Security, and Society, Furtwangen University, Furtwangen im Schwarzwald, Germany; Department of Psychology, University of Konstanz, Konstanz, Germany; Institute for Lifespan Development, Family, & Culture, University of Luxembourg, Esch-sur-Alzette, Luxembourg; Department of Developmental and Educational Psychology, Faculty of Psychology, University of Vienna, Vienna, Austria; Network Aging Research, Heidelberg University, Heidelberg, Germany; Euromov, University of Montpellier, Montpellier, France; Department of Psychology, Humboldt University Berlin, Berlin, Germany; Department of Prevention Research and Social Medicine, Institute for Community Medicine, University of Greifswald, Greifswald, Germany; Department of Human Development and Family Studies, Colorado State University, Fort Collins, USA; Network Aging Research, Heidelberg University, Heidelberg, Germany

**Keywords:** Developmental outcomes, Healthy aging, Health-enhancing behaviors, Mental, physical, cognitive health, Self-perceptions of aging

## Abstract

Research related to subjective aging, which describes how individuals perceive, interpret, and evaluate their own aging, has substantially grown in the past 2 decades. Evidence from longitudinal studies shows that subjective aging predicts health, quality of life, and functioning in later life. However, the existing literature on successful aging has mostly neglected the role of subjective aging. This paper proposes an extended framework of successful aging linking subjective aging conceptually and empirically to Rowe and Kahn’s ((1997). Successful aging. *Gerontologist*, 37(4), 433–440) 3 original key criteria of successful aging (i.e., avoiding disease and disability, maintaining high cognitive and physical function, and engagement with life). A particular focus is placed on subjective aging as an antecedent of successful aging. A review of the empirical subjective aging literature shows that subjective aging concepts consistently predict all 3 of Rowe and Kahn’s criteria of successful aging. Mechanisms underlying these relations are discussed at 3 levels, namely psychological, behavioral, and physiological pathways. The proposed addition also takes into consideration the interconnections between subjective aging and successful aging throughout the life span and across historical time. Finally, we discuss the importance of facilitating successful aging through systematic interventions that support more positive views of aging at the individual and societal levels.

Rowe and Kahn’s MacArthur model of successful aging (Rowe & Kahn, 1997, 2015) is considered the most influential successful aging (SA) framework and uses three criteria to define SA: (1) Avoiding disease and disability; (2) maintaining high cognitive and physical function; and (3) maintaining meaningful engagement with life. Despite its popularity, over the past decades the MacArthur, model of SA has received several criticisms, including the missing life course perspective of the model (Stowe & Cooney, 2015), as well as the argument that people with one or more chronic illnesses and with some level of physical disability may nonetheless interpret their aging as successful (Martinson & Berridge, 2015). Indeed, according to the stringent criteria of the MacArthur model of SA and the sole reliance on objective indicators of SA, SA is present in only a small percentage of older adults, and even less in very old adults (typically below 5%; Tesch-Römer et al., 2022). On the other hand, moving from solely objective criteria to solely subjective evaluations of successful aging as outcomes may result in a lack of interindividual variation with very high numbers of older adults describing themselves as aging successfully (Depp & Jeste, 2006). In an attempt to avoid such extreme perspectives, we posit that it is crucial to investigate the full range of factors that contribute to SA and that psychological antecedents of SA, such as subjective aging, need more consideration. Such a position reflects recent insights in psychology showing the relevance of subjective aging for key developmental outcomes such as health and cognitive performance. Surprisingly, past work that has adopted Rowe and Kahn’s model of SA has mostly neglected the construct of subjective aging. Subjective aging is used in this paper as an umbrella term capturing individuals’ subjective experiences, beliefs, and evaluations of their own aging (Wurm et al., 2017). The most frequent concepts and measurement approaches examined in studies on subjective aging in relation to health-related outcomes are Felt Age, Attitude Toward Own Aging, Aging-related Cognitions, and Awareness of Age-related Change (Shrira et al., 2022; Westerhof et al., 2023). *Felt Age* describes how old people feel. *Attitude Toward Own Aging* comprises an explicit personal, unidimensional evaluation of age-related changes (Lawton, 1975). *Aging-Related Cognitions*, as measured by the Aging-related Cognitions Scales, are multidimensional and provide scores for ongoing personal development, as well as physical and social losses (Steverink et al., 2001; Wurm et al., 2007). *Awareness of Age-related Change* assumes that development always involves gains and losses and that individuals have an explicit awareness of how their lives have changed due to getting older (Diehl & Wahl, 2010).

It is important to acknowledge that there are conceptual differences between these concepts (Klusmann et al., 2020). For example, feeling younger is seen as psychologically distancing oneself from one’s “true” age and age peers. Higher scores on other indicators of subjective aging, such as Attitudes Toward Own Aging, Aging-related Cognitions, and Awareness of Age-related Change, instead do not imply distancing oneself from one’s own aging. These indicators are generally referred to as *self-perceptions of aging*. In Westerhof et al.’s (2023) meta-analysis of subjective aging and health, 53 out of the 107 included longitudinal studies relied on subjective age, whereas the rest used self-perception of aging scales, hence, in the context of longitudinal effects on health and cognitive functioning, both approaches have been used in similar frequency. Both approaches also exert a similar impact on health outcomes, in the range of small effect sizes. Therefore, for the remainder of the paper, we collectively refer to these concepts as “subjective aging” without additional differentiation among indicators.

It is also important to mention that the construct of subjective aging is conceptually different from but empirically related to age stereotypes and ageism. Whereas age stereotypes refer to how individuals perceive older adults as a group, subjective aging reflects a person’s *own* behavioral experiences of growing older. Negative age stereotypes and ageism have detrimental effects on health (Chang et al., 2020). Hence, the argument that subjective aging needs better consideration in the discourse on SA is further supported by the findings of the age stereotype and ageism literature. However, in this paper, we deliberately focus on subjective aging and its different operationalizations.

## Subjective Aging and Successful Aging: A Needed Conceptual Connection

Rowe and Kahn (2015) only tangentially elaborated on the connection between subjective aging and SA besides mentioning that previous critiques of their model “propose (…) greater attention to individuals’ perceptions of their own aging” (p. 593). We see an important gap here. In the following, we elaborate on conceptual reasons why subjective aging and SA need to be better connected and suggest an integrative conceptual framework.

To begin with conceptual reasons, Rowe and Kahn (2015) themselves have argued that SA should be seen as a goal-directed and lifelong process that presumes active involvement and human agency. Therefore, studying subjective aging and SA in tandem may provide valuable insights into how and to what extent individuals can become producers of their own development and aging. Subjective aging certainly is not the only, but definitely, one important factor that has an impact on those behaviors (e.g., physical activity), resources (e.g., self-efficacy), and self-regulatory processes (e.g., acceptance of changes) that, over the life span, may influence whether individuals meet Rowe and Kahn’s criteria for SA. A major reason for this argument is that the perception of getting older increasingly becomes a core element of individuals’ self-concept in later life (Diehl et al., 2014). Furthermore, subjective aging is influenced by societal views of older age, and these are generally negative. Hence, incorporating more positively toned subjective aging may be a core issue of what Rowe and Kahn (2015) described as the need for “reengineering core societal institutions” (p. 594). Finally, subjective aging may broaden the scope of interventions that promote SA (Diehl et al., 2023).

**Figure 1.**
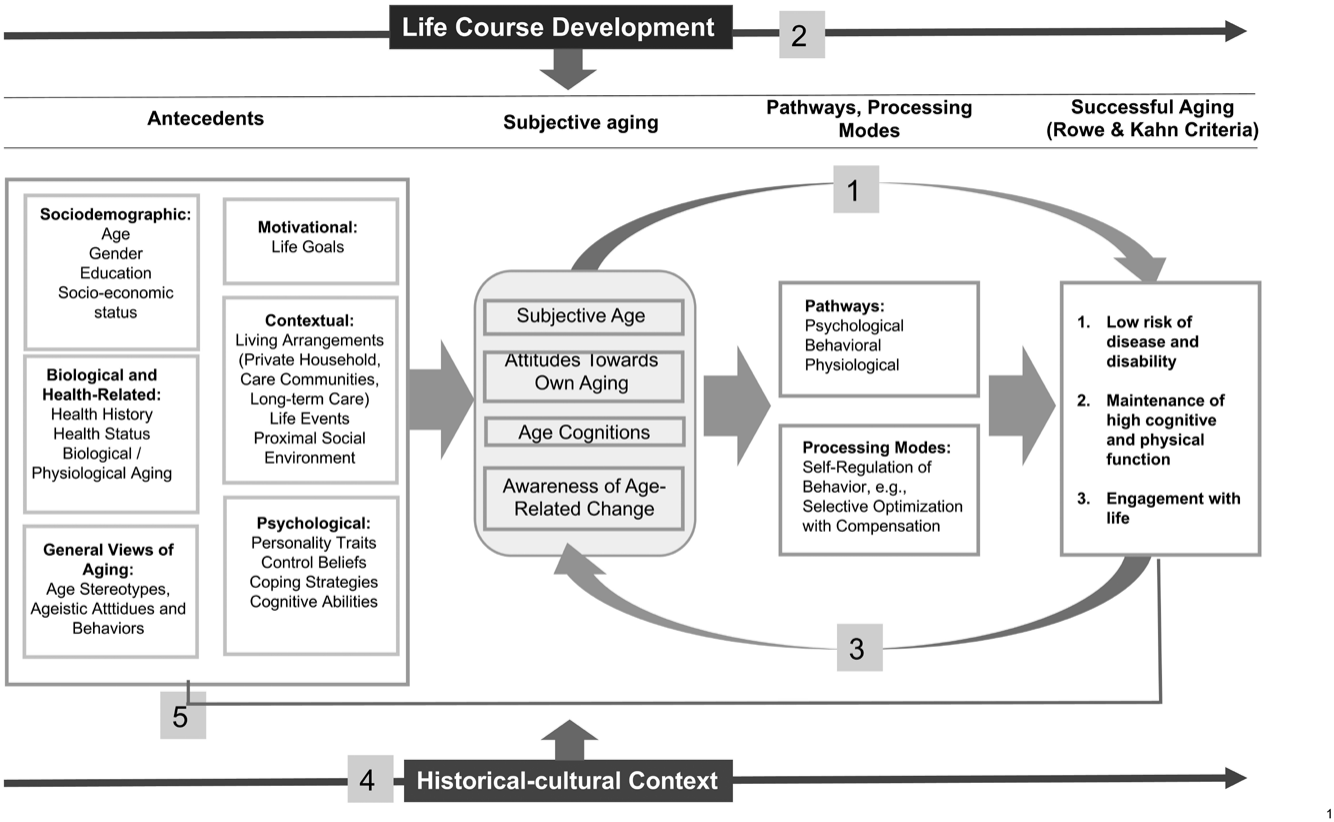
A model of subjective aging as a key factor for successful aging.

Figure 1 shows an extended conceptual framework of SA building on Rowe and Kahn’s (1997, 2015) criteria but with an explicit consideration of subjective aging.

The model acknowledges that several antecedents may influence subjective aging and SA. The effect of such antecedents on SA may be direct (e.g., health) or indirect through subjective aging (i.e., subjective aging as a mediator). Although the model visually gives prominence to subjective aging as a factor neglected in previous discourses on SA, it does not assume that its role for SA is more relevant than the role of other antecedents.

The model assumes that subjective aging has the potential to affect all three of Rowe and Kahn’s criteria of SA (point 1, Figure 1). In terms of pathways operating between subjective aging and SA outcomes (point 2, Figure 1), the model draws on Levy’s (2009) Stereotype Embodiment Theory, which postulates that subjective aging can influence health outcomes and mortality through three pathways, namely psychological, behavioral, and physiological pathways (see the section on “Pathways” for more details). Moreover, the model proposes a life span perspective (point 3, Figure 1) in how subjective aging unfolds and may differentially affect SA outcomes.

Importantly, the positive and negative objective changes that individuals may experience in the three criteria of SA are also assumed to influence their subjective aging; hence the model expects *bidirectionality* between subjective aging and SA (point 4, Figure 1). In parallel, historical-cultural contexts may shape subjective aging and therefore its effects on SA outcomes (point 5, Figure 1; Wettstein et al., 2023). Finally, the effects of subjective aging on SA may potentially be modified through interventions promoting positive self-perceptions of aging (point 6, Figure 1).

In the following, we aim to provide the strongest empirical evidence in support of the proposed framework, without claiming that we are providing a comprehensive review of the literature. At the same time, we aim to identify areas that are empirically less researched or show conflicting evidence, hence needing more empirical testing in the future.

## Subjective Aging as a Protective or Risk Factor of Successful Aging (Model, Point 1)

### Evidence Linking Subjective Aging to the Disease, Disability, and Physical Function-Related Facets of Successful Aging

Positive subjective aging is consistently associated with a lower risk of mental and physical health conditions, such as depression, anxiety, rheumatism, cancer, and arthritis (Debreczeni & Bailey, 2021; Tully-Wilson et al., 2021; Westerhof et al., 2023), lower risk of disability, including lower risk of frailty, falls, and functional difficulties (e.g., ability to do groceries and prepare one’s own meal; Fundenberger et al., 2022; Li et al., 2021; Sabatini, Wahl, et al., 2023). More positive subjective aging is associated with better biological aging, as indicated by lower presence of inflammatory markers greater telomere length (McLachlan et al., 2020; Stephan, Sutin, Luchetti, & Terracciano, 2023), and faster walking speed (Stephan et al., 2015). These results are supported by a meta-analysis comprising more than 100 longitudinal studies with a median observational interval of approximately 5 years (Westerhof et al., 2023). Moreover, Levy et al. (2002) pointed to the effect of subjective aging on longevity being comparable to the effect of established risk factors such as smoking or obesity. To date, 20 longitudinal studies (such as Wurm & Schäfer, 2022), have shown that people with more positive SPA live longer than those with more negative SPA (Westerhof et al., 2023). The evidence that subjective aging is an important protective or risk factor for the SA domain of disease and disability is thus strong and consistent.

### Evidence Linking Subjective Aging to the Cognitive Function Facet of Successful Aging

More positive subjective aging is associated with better objectively assessed (Debreczeni & Bailey, 2021) and informant-rated (Stephan et al., 2021) cognitive functioning, and lower dementia risk (Siebert et al., 2018). In their systematic review of longitudinal research, Tully-Wilson et al. (2021) found that more negative subjective aging predicted lower cognitive functioning and greater incidence of dementia-related disorders in the range of small to medium effects. In summary, the link between subjective aging and cognitive performance is well established (Fernández-Ballbé et al., 2023).

### Evidence Linking Subjective Aging to the Engagement With Life Facet of Successful Aging

Compared to the two previous SA indicators, evidence supporting a link between subjective aging and *engagement with life* is more limited. More positive subjective aging has been associated with more frequent future attendance of formal events focusing on older adults (Schwartz et al., 2021), lower loneliness cross-sectionally (Xie et al., 2022), and greater future engagement with social (Shoushtari-Moghaddam et al., 2022) and leisure activities (Bu et al., 2023). Moreover, bidirectional associations have been found between positive subjective aging and productive activities, including volunteering (Huo et al., 2020), provision of advice and emotional support to others (Schwartz et al., 2021), and engagement in political activities (Shoushtari-Moghaddam et al., 2022). More positive subjective aging has also been found cross-sectionally associated with caring for grandchildren (Bordone & Arpino, 2015). Thus, there is emerging evidence showing that subjective aging is also relevant for promoting engagement with life as the third major facet of SA.

## Pathways From Subjective Aging to Successful Aging (Model, Point 2)

As shown in Figure 1, subjective aging may affect all three of Rowe and Kahn’s criteria of SA through psychological, behavioral, and physiological pathways (Levy, 2009). All three pathways are supported by evidence. The psychological pathway captures how more positive subjective aging may increase self-efficacy, adaptive self-regulation, will to live, and a positive outlook toward the future (Dutt & Wahl, 2018; Levy et al., 2002; Wurm et al., 2013). The behavioral pathway reflects how subjective aging influences engagement in health-enhancing behaviors and adaptive behaviors (Dutt et al., 2016; Sun & Smith, 2017). Last, the physiological pathway captures how negative subjective aging may lead to negative biological processes and, consequently, to health-related conditions (Schönstein et al., 2022; Stephan, Sutin, Luchetti, & Terracciano, 2023).

## A Lifespan Perspective on Bidirectional Associations Between Subjective Aging and Successful Aging (Model, Points 3 and 4)

Discourses on subjective aging and SA may benefit from adopting a life span perspective for several reasons (Stowe & Cooney, 2015). First, both individuals’ subjective aging and SA likely change over time, which is not surprising given the dynamic nature of aging processes. Importantly, empirical evidence suggests that more negative and loss-oriented self-views increase with aging (Kornadt et al., 2016). Still, subjective aging is malleable and subject to change across the life span. Heterogeneity in self-perceptions of one’s present and future aging is particularly pronounced in midlife (Miche et al., 2014). Variables explaining such heterogeneity include personality factors, beginning signs of one’s aging (Kornadt et al., 2019), and critical life events (Rupprecht et al., 2022; Turner et al., 2023).

Second, life span patterns of subjective aging and SA are very likely intertwined, and the association between subjective aging and SA throughout the life span is very likely bidirectional. Indeed, the positive and negative objective changes that individuals may experience in the three criteria of SA seem to also influence their subjective aging. Empirical evidence shows that increases in depressive and anxiety symptoms (Sabatini et al., 2022), newly diagnosed health conditions (e.g., cancer and cardiovascular events; Schönstein, Dallmeier, et al., 2021; Wurm et al., 2020), and greater functional difficulties (Kaspar et al., 2022) all lead to more negative subjective aging. Bidirectionality might indeed be a critical element of maintaining or regaining SA. Specifically, the interplay between subjective aging and SA might end up in a downward spiral, in which decreasing health may lead to more negative subjective aging. This, in turn, may undermine the motivation for training, exercise, intervention, or rehabilitation as well as increase physiological risk constellations, resulting in poorer SA outcomes.

Third, a life span perspective provides opportunities to start optimizing SA from young adulthood and midlife. Indeed, according to Stereotype Embodiment Theory (Levy, 2009), individuals form age-related beliefs early in childhood and these become internalized later in life and influence how individuals perceive their own aging throughout their life span (Levy, 2009). Recent evidence suggests that subjective experiences of aging are relevant and related to health outcomes starting in young adulthood (Sabatini, Rupprecht, et al., 2023).

Fourth, the mechanisms linking subjective aging with SA might be different at different stages of the life span (Kornadt et al., 2020). For example, although “continued engagement with life” might be increasingly driven by health constraints (and less so by subjective aging) at older ages, subjective aging can help to prevent attributing health constraints to aging. Hence, subjective aging can buffer the detrimental effects of health events on life engagement and the effects of giving up volunteering on life satisfaction (Huo & Kim, 2022). Subjective aging might also become more self-relevant (Levy, 2009) and important at older ages for other SA outcomes as it may help to compensate for loss-related changes (Baltes, 1997).

Overall, subjective aging processes happening across individuals’ lifetimes may have the potential to enrich Rowe and Kahn’s (2015) emphasis on the role of the life course for SA.

## Historical–Cultural Impact on Subjective Aging: Implications for Successful Aging (Model, Point 5)

Subjective aging may change across historical, secular, and cultural contexts (see point 4, Figure 1; Ackerman & Chopik, 2020). For example, Wettstein et al. (2023) found that when investigating secular change in subjective age across 25 years by comparing birth cohorts of middle-aged and older adults who had similar ages and were assessed at different points in time, the later-born cohorts felt younger than the earlier-born. Those from more recent cohorts also maintained feeling younger longer into advanced old age. More research is needed to better understand whether historical change (i.e., changes linked to historical events such as growing up during World War II) and secular change (i.e., changes linked to growing up in different environmental conditions) impact - how SA and subjective aging are interrelated. Furthermore, shared cultural influences certainly affect subjective aging, although existing data are limited. For example, adults aged 40 years and older in Burkina Faso, a country in West Africa, felt younger to a lesser extent than their counterparts in a Western sample (Schönstein, Ngo, et al., 2021).

Subjective (and societal) views on aging may provide an important springboard for models of SA to consider whether and how the societal perception of what is SA varies across historical and cultural contexts. For example, North and Fiske (2015) replicated in their meta-analysis not only the previously established finding that increases in population aging significantly predicted negative attitudes toward older adults. They also found that cultural *individualism* typical for Western countries significantly predicted positive attitudes toward older adults, whereas the collectivist traditions that exist in rapidly aging Eastern societies did not have the same effect.

## Subjective Aging as an Intervention Target to Promote Successful Aging (Model, Point 6)

Ultimately, subjective aging may provide an avenue to optimize SA; that is, promoting positive subjective aging might help to avoid as much as possible disease and disability, maintain high cognitive and physical functioning, and sustain engagement with life (Rowe & Kahn, 1997).

Several interventions have been developed to change middle-aged and older adults’ SPA and consequently developmental outcomes (Beyer et al., 2019; Diehl et al., 2023; Wolff et al., 2014). These interventions have used different strategies to increase positive subjective aging. Some interventions indirectly targeted age stereotypes and subjective aging through promotion of health-enhancing behaviors (e.g., physical activity and hence providing mastery experiences; Beyer et al., 2019). Others targeted both subjective aging and health-related behaviors simultaneously (e.g., Diehl et al., 2023). These interventions typically focus on self-perceptions of aging, and not felt age. They showed that the promotion of positive, yet realistic subjective aging is possible and effective even among older adults in poor physical health. However, a major gap in this area of inquiry concerns the long-term maintenance of the observed effects. Another unanswered question is whether successful behavior changes, such as increased engagement in physical activity or eating a healthier diet, reinforce the maintenance of more positive subjective and successful aging.

Interventions that either actively (e.g., rehabilitative programs; Crocker et al., 2013) or passively (e.g., self-compassion programs promoting acceptance of age-related challenges and conditions; Toise et al., 2014) promote adaptation to age-related changes may also have an effect on subjective aging. Conversely, interventions targeting healthy/successful aging should, by definition, target diverse and constructive views on aging, given the strong evidence of their health-promoting role throughout the life span (Klusmann et al., 2021).

## Concluding Remarks and Future Directions

Throughout the life span, individuals’ interpretations of their own aging make a profound difference in everyday life and whether and how existing opportunity structures are used as developmental impulses for one’s own life (Diehl & Wahl, 2010). In other words, subjective views of one’s aging play an important role in infusing agency toward SA as well as strengthening the motivation to invest, for example, in health maintenance, prevention, rehabilitation, training, and social connectedness. Therefore, including subjective aging in models of SA seems an important step forward for the implementation of current and future strategies and interventions promoting SA through the increase of positive subjective aging.

We hope that the suggested conceptual framework may guide future research toward closing several empirical gaps. First, the impact of subjective aging on engagement with life as a major sociobehavioral outcome of SA as well as mechanisms underlying this affect needs more research. A major issue is that subjective aging research in general needs more social contextualization and, thus, overcomes its current centeredness on the individual as the unit of analysis (Chu et al., 2020; Rupprecht et al., 2022).

Second, interdisciplinary research, particularly collaborations between the behavioral and biosciences of aging, is needed to better understand the pathways underlying the associations between subjective aging and SA, and their interplay. More efforts are needed for example to examine how various biomarkers act in parallel as well as whether their associations with subjective aging depend on the specific subjective aging indicator considered, thus moving away from the approach of evaluating only a single biomarker or a single subjective aging operationalization. For example, Stephan, Sutin, Luchetti, Aschwanden, et al. (2023) found that poorer cognitive functioning (memory) was partially mediated through higher fasting glucose, higher cystatin C, higher NT-proBNP, and accelerated epigenetic aging. In contrast, more negative attitudes toward aging were related to worse memory through lower Vitamin D3, higher fasting glucose, higher cystatin C, higher NT-proBNP, and accelerated epigenetic aging.

Third, there is the need to investigate subjective aging as a moderator in the associations between risk factors and Rowe and Kahn’s indicators of SA. Indeed, it is currently mostly unknown to what extent positive subjective aging can “eliminate” or buffer the detrimental influence of other risk factors on SA. Best confirmed currently seems the investment in health behaviors, preventive action, and physical activity (Diehl et al., 2023; Westerhof et al., 2023). However, the interplay between subjective aging and other risk factors such as stress, obesity, substance use, or treatment adherence is less clear.

Fourth, future research needs to intensify the investigation of the potential bidirectional subjective aging-successful aging interplay. Importantly, the bidirectional association between subjective aging and SA should be investigated from a life span perspective. That is, there is a need to understand whether negative subjective aging in young adulthood or midlife increases the risk of “non-successful aging” in late life. Indeed, most of the available evidence focuses on the effect of subjective aging on SA in the second half of life. How physiological pathways operate in a bidirectional way between subjective aging and SA outcomes also needs to be understood in a better way and longitudinal studies are needed to address this topic (Schönstein et al., 2022).

Fifth, although numerous studies support the links between perceived negative age-related changes (i.e., negative subjective aging) and lack of SA, evidence on whether perceived positive age-related changes (i.e., positive subjective aging) can attenuate the detrimental impact of negative subjective aging on SA is scarce (Diehl et al., 2021). Future studies should investigate the interplay between positive and negative subjective aging as a resilience or risk factor for successful aging.

Sixth, a better understanding of the historical-cultural embeddedness of subjective aging and SA may help to recognize the historical and cultural relativity and diversity of SA.

In conclusion, we hope to have shown that both from a conceptual and an empirical perspective, subjective aging deserves more consideration as an important life-long antecedent of SA.

## Data Availability

The authors do not report data and therefore the preregistration and data availability requirements are not applicable.

## References

[CIT0001] Ackerman, L. S., & Chopik, W. J. (2020). Cross-cultural comparisons in implicit and explicit age bias. Personality and Social Psychology Bulletin, 47(6), 953–968. https://doi.org/10.1177/0146167220950070 32875949

[CIT0002] Baltes, P. B. (1997). On the incomplete architecture of human ontogeny: Selection, optimization, and compensation as foundation of developmental theory. American Psychologist, 52(4), 366–380. https://doi.org/10.1037//0003-066x.52.4.366 9109347

[CIT0003] Beyer, A. K., Wolff, J. K., Freiberger, E., & Wurm, S. (2019). Are self-perceptions of ageing modifiable? Examination of an exercise programme with vs. without a self-perceptions of ageing-intervention for older adults. Psychology & Health, 34(6), 661–676. https://doi.org/10.1080/08870446.2018.1556273 30628483

[CIT0004] Bordone, V., & Arpino, B. (2015). Do grandchildren influence how old you feel? Journal of Aging and Health, 28(6), 1055–1072. https://doi.org/10.1177/0898264315618920 26656157

[CIT0005] Bu, F., Mak, H. W., Bone, J. K., Gao, Q., Sonke, J. K., & Fancourt, D. (2023). Leisure engagement and self-perceptions of aging: Longitudinal analysis of concurrent and lagged relationships. Journals of Gerontology, Series B: Psychological Sciences and Social Sciences, 79, gbad182. https://doi.org/10.1093/geronb/gbad182 PMC10873860 38134236

[CIT0006] Chang, E.-S., Kannoth, S., Levy, S., Wang, S.-Y., Lee, J. E., & Levy, B. R. (2020). Global reach of ageism on older persons’ health: A systematic review. PLoS One, 15(1), e0220857. https://doi.org/10.1371/journal.pone.0220857 31940338 PMC6961830

[CIT0007] Chu, L., Lay, J. C., Tsang, V. H. L., & Fung, H. H. (2020). Attitudes toward aging: A glance back at research developments over the past 75 years. Journals of Gerontology, Series B: Psychological Sciences and Social Sciences, 75(6), 1125–1129. https://doi.org/10.1093/geronb/gbz155 32484890

[CIT0008] Crocker, T., Young, J., Forster, A., Brown, L., Ozer, S., & Greenwood, D. C. (2013). The effect of physical rehabilitation on activities of daily living in older residents of long-term care facilities: Systematic review with meta-analysis. Age and Ageing, 42(6), 682–688. https://doi.org/10.1093/ageing/aft133 24004604

[CIT0009] Debreczeni, A. F., & Bailey, P. E. (2021). A systematic review and meta-analysis of subjective age and the association with cognition, subjective well-being, and depression. Journals of Gerontology, Series B: Psychological Sciences and Social Sciences, 76(3), 471–482. https://doi.org/10.1093/geronb/gbaa069 32453828

[CIT0010] Depp, C. A., & Jeste, D. V. (2006). Definitions and predictors of successful aging: A comprehensive review of larger quantitative studies. American Journal of Geriatric Psychiatry: Official Journal of the American Association for Geriatric Psychiatry, 14(1), 6–20. https://doi.org/10.1097/01.JGP.0000192501.03069.bc 16407577

[CIT0011] Diehl, M., Rebok, G. W., Roth, D. L., Nehrkorn-Bailey, A., Rodriguez, D., Tseng, H.-Y., & Chen, D. (2023). Examining the malleability of negative views of aging, self-efficacy beliefs, and behavioral intentions in middle-aged and older adults. Journals of Gerontology, Series B: Psychological Sciences and Social Sciences, 78, 2009–2020. https://doi.org/10.1093/geronb/gbad130 37718651 PMC10699749

[CIT0012] Diehl, M. K., Brothers, A. F., & Wahl, H.-W. (2021). Self-perceptions and awareness of aging: Past, present, and future. In K. W. Schaie & S. L. Willis (Eds.), Handbook of the psychology of aging (8th ed.). Elsevier. https://doi.org/10.1016/B978-0-12-816094-7.00001-5

[CIT0013] Diehl, M. K., & Wahl, H.-W. (2010). Awareness of age-related change: Examination of a (mostly) unexplored concept. Journals of Gerontology, Series B: Psychological Sciences and Social Sciences, 65B(3), 340–350. https://doi.org/10.1093/geronb/gbp110 20008026 PMC2853600

[CIT0014] Diehl, M. K., Wahl, H.-W., Barrett, A. E., Brothers, A. F., Miche, M., Montepare, J. M., Westerhof, G. J., & Wurm, S. (2014). Awareness of aging: Theoretical considerations on an emerging concept. Developmental Review, 34(2), 93–113. https://doi.org/10.1016/j.dr.2014.01.001 24958998 PMC4064469

[CIT0015] Dutt, A. J., Gabrian, M., & Wahl, H.-W. (2016). Awareness of age-related change and depressive symptoms in middle and late adulthood: Longitudinal associations and the role of self-regulation and calendar age. Journals of Gerontology, Series B: Psychological Sciences and Social Sciences, 73(6), gbw095–953. https://doi.org/10.1093/geronb/gbw095 27534425

[CIT0016] Dutt, A. J., & Wahl, H.-W. (2018). Future time perspective and general self-efficacy mediate the association between awareness of age-related losses and depressive symptoms. European Journal of Ageing, 16(2), 227–236. https://doi.org/10.1007/s10433-018-0482-3 31139036 PMC6509274

[CIT0017] Fernández-Ballbé, O., Martin-Moratinos, M., Saiz, J., Gallardo-Peralta, L., & Barrón López de Roda, A. (2023). The relationship between subjective aging and cognition in elderly people: A systematic review. Healthcare, 11(24), 3115. https://doi.org/10.3390/healthcare11243115 38132005 PMC10743019

[CIT0018] Fundenberger, H., Stephan, Y., Terracciano, A., Dupré, C., Bongue, B., Hupin, D., Barth, N., & Canada, B. (2022). Subjective age and falls in older age: Evidence from two longitudinal cohorts. Journals of Gerontology, Series B: Psychological Sciences and Social Sciences, 77(10), 1814–1819. https://doi.org/10.1093/geronb/gbac094 35861191 PMC9535769

[CIT0019] Huo, M., & Kim, K. (2022). Volunteering dynamics and life satisfaction: Self-perceptions of aging as a buffer. Journals of Gerontology, Series B: Psychological Sciences and Social Sciences, 77(2), 321–331. https://doi.org/10.1093/geronb/gbab104 34115861

[CIT0020] Huo, M., Miller, L. M. S., Kim, K., & Liu, S. (2020). Volunteering, self-perceptions of aging, and mental health in later life. Gerontologist, 61, 1131–1140. https://doi.org/10.1093/geront/gnaa164 33103726

[CIT0021] Kaspar, R., Wahl, H.-W., Diehl, M. K., & Zank, S. (2022). Subjective views of aging in very old age: Predictors of 2-year change in gains and losses. Psychology and Aging, 37(4), 503–516. https://doi.org/10.1037/pag0000684 35467913 PMC10026176

[CIT0022] Klusmann, V., Gow, A. J., Robert, P., & Oettingen, G. (2021). Using theories of behavior change to develop interventions for healthy aging. Journals of Gerontology, Series B: Psychological Sciences and Social Sciences, 76(Suppl_2), S191–S205. https://doi.org/10.1093/geronb/gbab111 34515775

[CIT0023] Klusmann, V., Notthoff, N., Beyer, A.-K., Blawert, A., & Gabrian, M. (2020). The assessment of views on ageing: A review of self-report measures and innovative extensions. European Journal of Ageing, 17, 403–433. https://doi.org/10.1007/s10433-020-00556-9 33376461 PMC7752934

[CIT0024] Kornadt, A. E., Hess, T. M., Voss, P., & Rothermund, K. (2016). Subjective age across the life span: A differentiated, longitudinal approach. Journals of Gerontology, Series B: Psychological Sciences and Social Sciences, 73(5), gbw072–777. https://doi.org/10.1093/geronb/gbw072 27334638

[CIT0025] Kornadt, A. E., Kessler, E.-M., Wurm, S., Bowen, C. E., Gabrian, M., & Klusmann, V. (2020). Views on ageing: A lifespan perspective. European Journal of Ageing, 17(4), 387–401. https://doi.org/10.1007/s10433-019-00535-9 33380996 PMC7752932

[CIT0026] Kornadt, A. E., Siebert, J. S., & Wahl, H.-W. (2019). The interplay of personality and attitudes toward own aging across two decades of later life. PLoS One, 14(10), e0223622. https://doi.org/10.1371/journal.pone.0223622 31596876 PMC6785129

[CIT0027] Lawton, M. P. (1975). The Philadelphia Geriatric Center Morale Scale: A revision. Journal of Gerontology, 30(1), 85–89. https://doi.org/10.1093/geronj/30.1.85 1109399

[CIT0028] Levy, B. R. (2009). Stereotype embodiment: A psychosocial approach to aging. Current Directions in Psychological Science, 18(6), 332–336. https://doi.org/10.1111/j.1467-8721.2009.01662.x 20802838 PMC2927354

[CIT0029] Levy, B. R., Slade, M. D., Kunkel, S. R., & Kasl, S. V. (2002). Longevity increased by positive self-perceptions of aging. Journal of Personality and Social Psychology, 83(2), 261–270. https://doi.org/10.1037//0022-3514.83.2.261 12150226

[CIT0030] Li, Y., Liu, M., Miyawaki, C. E., Sun, X., Hou, T., Tang, S., & Szanton, S. L. (2021). Bidirectional relationship between subjective age and frailty: A prospective cohort study. BMC Geriatrics, 21(1), 1–9. https://doi.org/10.1186/s12877-021-02344-1 34187378 PMC8244193

[CIT0031] Martinson, M., & Berridge, C. (2015). Successful aging and its discontents: A systematic review of the social gerontology literature. Gerontologist, 55(1), 58–69. https://doi.org/10.1093/geront/gnu037 24814830 PMC4986586

[CIT0032] McLachlan, K. J. J., Cole, J. H., Harris, S. E., Marioni, R. E., Deary, I. J., & Gale, C. R. (2020). Attitudes to ageing, biomarkers of ageing and mortality: The Lothian Birth Cohort 1936. Journal of Epidemiology and Community Health, 74(4), 377–383. https://doi.org/10.1136/jech-2019-213462 31992610 PMC7079194

[CIT0033] Miche, M., Elsässer, V. C., Schilling, O. K., & Wahl, H.-W. (2014). Attitude toward own aging in midlife and early old age over a 12-year period: Examination of measurement equivalence and developmental trajectories. Psychology and Aging, 29(3), 588–600. https://doi.org/10.1037/a0037259 25244478

[CIT0034] North, M. S., & Fiske, S. T. (2015). Modern attitudes toward older adults in the aging world: A cross-cultural meta-analysis. Psychological Bulletin, 141(5), 993–1021. https://doi.org/10.1037/a0039469 26191955

[CIT0035] Rowe, J. W., & Kahn, R. L. (1997). Successful aging. Gerontologist, 37(4), 433–440. https://doi.org/10.1093/geront/37.4.433 9279031

[CIT0036] Rowe, J. W., & Kahn, R. L. (2015). Successful aging 2.0: Conceptual expansions for the 21st century. Journals of Gerontology, Series B: Psychological Sciences and Social Sciences, 70, 593–596. https://doi.org/10.1093/geronb/gbv025 25878054

[CIT0037] Rupprecht, F. S., Sabatini, S., Diehl, M. K., Gerstorf, D., Kaspar, R., Schilling, O. K., & Wahl, H.-W. (2022). Awareness of age-related change in the context of major life events. Frontiers in Psychiatry, 13, 954048. https://doi.org/10.3389/fpsyt.2022.954048 36386972 PMC9650375

[CIT0039] Sabatini, S., Siebert, J. S., Diehl, M. K., Brothers, A., & Wahl, H.-W. (2022). Identifying predictors of self-perceptions of aging based on a range of cognitive, physical, and mental health indicators: Twenty-year longitudinal findings from the ILSE study. Psychology and Aging, 37(4), 486–502. https://doi.org/10.1037/pag0000668 34941356 PMC10413976

[CIT0038] Sabatini, S., Rupprecht, F., Diehl, M., Wahl, H.-W., Kaspar, R., Schilling, O., & Gerstorf, D. (2023). Levels of awareness of age-related gains and losses throughout adulthood and their developmental correlates. Psychology and Aging, 38(8), 837–853. https://doi.org/10.1037/pag0000784 37902673

[CIT0040] Sabatini, S., Wahl, H.-W., Diehl, M., Clare, L., Ballard, C., Brooker, H., Corbett, A., Hampshire, A., & Stephan, B. C. M. (2023). Testing bidirectionality in associations of awareness of age-related gains and losses with physical, mental, and cognitive functioning across one year: The role of age. Journals of Gerontology, Series B: Psychological Sciences and Social Sciences, 78(12), 2026–2036. https://doi.org/10.1093/geronb/gbad150 37801677 PMC10699739

[CIT0041] Schönstein, A., Dallmeier, D., Denkinger, M., Rothenbacher, D., Klenk, J., Bahrmann, A., & Wahl, H.-W. (2021). Health and subjective views on aging: Longitudinal findings from the ActiFE Ulm Study. Journals of Gerontology, Series B: Psychological Sciences and Social Sciences, 76(7), 1349–1359. https://doi.org/10.1093/geronb/gbab023 33528511 PMC8363042

[CIT0042] Schönstein, A., Ngo, D. T. T., Stephan, Y., Siè, A., Harling, G., Bärnighausen, T., & Wahl, H.-W. (2021). Feeling younger in rural Burkina Faso: Exploring the role of subjective age in the light of previous research from high-income countries. Journals of Gerontology, Series B: Psychological Sciences and Social Sciences, 76(10), 2029–2040. https://doi.org/10.1093/geronb/gbab151 34379769 PMC8599075

[CIT0043] Schönstein, A., Trares, K., & Wahl, H.-W. (2022). Subjective views of aging and objective aging biomarkers: Achievements and questions in an emerging research area. In Y. Palgi (Ed.), Subjective views of aging: Theory, research, and practice (pp. 153–168). Springer Nature. https://doi.org/10.1007/978-3-031-11073-3_9

[CIT0044] Schwartz, E., Ayalon, L., & Huxhold, O. (2021). Exploring the reciprocal associations of perceptions of aging and social involvement. Journals of Gerontology, Series B: Psychological Sciences and Social Sciences, 76(3), 563–573. https://doi.org/10.1093/geronb/gbaa008 31950185

[CIT0045] Shoushtari-Moghaddam, E., Kaveh, M. H., & Nazari, M. (2022). Ageing perception and social functioning in older adults: A narrative review. Working with Older People, 26, 165–173. https://doi.org/10.1108/wwop-09-2021-0051

[CIT0046] Shrira, A., Palgi, Y., & Diehl, M. (2022). Advancing the field of subjective views of aging: An overview of recent achievements. In Y. Palgi, A. Shrira, & M. Diehl (Eds.), Subjective views of aging: Theory, research, and practice (pp. 11–37). https://doi.org/10.1007/978-3-031-11073-3_2

[CIT0047] Siebert, J. S., Wahl, H.-W., Degen, C., & Schröder, J. (2018). Attitude toward own aging as a risk factor for cognitive disorder in old age: 12-year evidence from the ILSE study. Psychology and Aging, 33(3), 461–472. https://doi.org/10.1037/pag0000252 29756803

[CIT0049] Stephan, Y., Sutin, A. R., Luchetti, M., & Terracciano, A. (2021). Subjective age and informant-rated cognition and function: A prospective study. Psychology and Aging, 36(3), 338–343. https://doi.org/10.1037/pag0000566 32790457

[CIT0048] Stephan, Y., Sutin, A. R., Luchetti, M., Aschwanden, D., & Terracciano, A. (2023). The mediating role of biomarkers in the association between subjective aging and episodic memory. Journals of Gerontology, Series B: Psychological Sciences and Social Sciences, 78(2), 242–252. https://doi.org/10.1093/geronb/gbac155 36179098 PMC9938926

[CIT0050] Stephan, Y., Sutin, A. R., Luchetti, M., & Terracciano, A. (2023). The prospective relationship between subjective aging and inflammation: Evidence from the health and retirement study. Psychophysiology, 60(2), e14177. https://doi.org/10.1111/psyp.14177 36124383

[CIT0051] Stephan, Y., Sutin, A. R., & Terracciano, A. (2015). “Feeling younger, walking faster”: Subjective age and walking speed in older adults. Age, 37, 1–12. https://doi.org/10.1007/s11357-015-9830-9 26296609 PMC5005834

[CIT0052] Steverink, N., Westerhof, G. J., Bode, C., & Dittmann-Kohli, F. (2001). The personal experience of aging, individual resources, and subjective well-being. Journals of Gerontology, Series B: Psychological Sciences and Social Sciences, 56(6), P364–P373. https://doi.org/10.1093/geronb/56.6.p364 11682590

[CIT0053] Stowe, J. D., & Cooney, T. M. (2015). Examining Rowe and Kahn’s concept of successful aging: Importance of taking a life course perspective. Gerontologist, 55(1), 43–50. https://doi.org/10.1093/geront/gnu055 24906516 PMC4986588

[CIT0054] Sun, J. K., & Smith, J. (2017). Self-perceptions of aging and perceived barriers to care: Reasons for health care delay. Gerontologist, 57(Suppl_2), S216–S226. https://doi.org/10.1093/geront/gnx014 28854604 PMC5881713

[CIT0055] Tesch-Römer, C., Wahl, H.-W., Rattan, S. I. S., & Ayalon, L. (2022). Successful ageing: Ambition and ambivalence. Oxford University Press. https://doi.org/10.1093/med/9780192897534.001.0001

[CIT0056] Toise, S. C. F., Sears, S. F., Schoenfeld, M. H., Blitzer, M. L., Marieb, M. A., Drury, J. H., Slade, M. D., & Donohue, T. J. (2014). Psychosocial and cardiac outcomes of yoga for ICD patients: A randomized clinical control trial. Pacing and Clinical Electrophysiology, 37(1), 48–62. https://doi.org/10.1111/pace.12252 23981048 PMC4524735

[CIT0057] Tully-Wilson, C., Bojack, R., Millear, P. M., Stallman, H. M., Allen, A., & Mason, J. (2021). Self-perceptions of aging: A systematic review of longitudinal studies. Psychology and Aging, 36(7), 773–789. https://doi.org/10.1037/pag0000638 34498894

[CIT0058] Turner, S. G., Witzel, D. D., Stawski, R. S., & Hooker, K. (2023). How do marital transitions affect self-perceptions of aging? Research on Aging, 45(3–4), 374–384. https://doi.org/10.1177/01640275221113219 35815741 PMC10426280

[CIT0059] Westerhof, G. J., Nehrkorn-Bailey, A. M., Tseng, H.-Y., Brothers, A., Siebert, J. S., Wurm, S., Wahl, H.-W., & Diehl, M. (2023). Longitudinal effects of subjective aging on health and longevity: An updated meta-analysis. Psychology and Aging, 38(3), 147–166. https://doi.org/10.1037/pag0000737 36972091 PMC10192139

[CIT0060] Wettstein, M., Wahl, H.-W., Drewelies, J., Wurm, S., Huxhold, O., Ram, N., & Gerstorf, D. (2023). Younger than ever? Subjective age is becoming younger and remains more stable in middle-aged and older adults today. Psychological Science, 34(6), 647–656. https://doi.org/10.1177/09567976231164553 37071708

[CIT0061] Wolff, J. K., Warner, L. M., Ziegelmann, J. P., & Wurm, S. (2014). What do targeting positive views on ageing add to a physical activity intervention in older adults? Results from a randomised controlled trial [Psychol Health]. Psychology & Health, 29(8), 915–932. https://doi.org/10.1080/08870446.2014.896464 24559210

[CIT0062] Wurm, S., Diehl, M., Kornadt, A. E., Westerhof, G. J., & Wahl, H.-W. (2017). How do views on aging affect health outcomes in adulthood and late life? Explanations for an established connection. Developmental Review, 46, 27–43. https://doi.org/10.1016/j.dr.2017.08.002 33927468 PMC8081396

[CIT0063] Wurm, S., & Schäfer, S. K. (2022). Gain-but not loss-related self-perceptions of aging predict mortality over a period of 23 years: A multidimensional approach. Journal of Personality and Social Psychology, 123, 636–653. https://doi.org/10.1037/pspp0000412 35201819

[CIT0064] Wurm, S., Tesch-Römer, C., & Tomasik, M. J. (2007). Longitudinal findings on aging-related cognitions, control beliefs, and health in later life. Journals of Gerontology, Series B: Psychological Sciences and Social Sciences, 62(3), P156–P164. https://doi.org/10.1093/geronb/62.3.p156 17507583

[CIT0065] Wurm, S., Warner, L. M., Ziegelmann, J. P., Wolff, J. K., & Schuz, B. (2013). How do negative self-perceptions of aging become a self-fulfilling prophecy? Psychology and Aging, 28(4), 1088–1097. https://doi.org/10.1037/a0032845 24128074

[CIT0066] Wurm, S., Wiest, M., Wolff, J. K., Beyer, A.-K., & Spuling, S. M. (2020). Changes in views on aging in later adulthood: The role of cardiovascular events. European Journal of Ageing, 17, 457–467. https://doi.org/10.1007/s10433-019-00547-5 33380999 PMC7752931

[CIT0067] Xie, J., Zhang, B., Yao, Z., Zhang, W., Wang, J., Zhao, C.-n, & Huang, X. (2022). The effect of subjective age on loneliness in the old adults: The chain mediating role of resilience and self-esteem. Frontiers in Public Health, 10, 907934. https://doi.org/10.3389/fpubh.2022.907934 35983360 PMC9379278

